# Validation of the Regicor Short Physical Activity Questionnaire for the Adult Population

**DOI:** 10.1371/journal.pone.0168148

**Published:** 2017-01-13

**Authors:** Luis Molina, Manuel Sarmiento, Judith Peñafiel, David Donaire, Judith Garcia-Aymerich, Miquel Gomez, Mireia Ble, Sonia Ruiz, Albert Frances, Helmut Schröder, Jaume Marrugat, Roberto Elosua

**Affiliations:** 1 Cardiology Department, Hospital del Mar, Barcelona, Spain; 2 IMIM (Hospital del Mar Medical Research Institute), Barcelona, Spain; 3 Gerencia de Atención Primaria de Mallorca – Ibsalut, Mallorca, Spain; 4 ISGlobal, Centre for Research in Environmental Epidemiology (CREAL), Barcelona, Spain; 5 Universitat Pompeu Fabra (UPF), Barcelona, Spain; 6 CIBER Epidemiología y Salud Pública (CIBERESP), Barcelona, Spain; Universidad Europea de Madrid, SPAIN

## Abstract

**Objective:**

To develop and validate a short questionnaire to estimate physical activity (PA) practice and sedentary behavior for the adult population.

**Methods:**

The short questionnaire was developed using data from a cross-sectional population-based survey (n = 6352) that included the Minnesota leisure-time PA questionnaire. Activities that explained a significant proportion of the variability of population PA practice were identified. Validation of the short questionnaire included a cross-sectional component to assess validity with respect to the data collected by accelerometers and a longitudinal component to assess reliability and sensitivity to detect changes (n = 114, aged 35 to 74 years).

**Results:**

Six types of activities that accounted for 87% of population variability in PA estimated with the Minnesota questionnaire were selected. The short questionnaire estimates energy expenditure in total PA and by intensity (light, moderate, vigorous), and includes 2 questions about sedentary behavior and a question about occupational PA. The short questionnaire showed high reliability, with intraclass correlation coefficients ranging between 0.79 to 0.95. The Spearman correlation coefficients between estimated energy expenditure obtained with the questionnaire and the number of steps detected by the accelerometer were as follows: 0.36 for total PA, 0.40 for moderate intensity, and 0.26 for vigorous intensity. The questionnaire was sensitive to detect changes in moderate and vigorous PA (correlation coefficients ranging from 0.26 to 0.34).

**Conclusion:**

The REGICOR short questionnaire is reliable, valid, and sensitive to detect changes in moderate and vigorous PA. This questionnaire could be used in daily clinical practice and epidemiological studies.

## Introduction

Physical activity (PA) is associated with a lower rate of various chronic diseases and premature death [1], and inactivity is considered an independent risk factor for several chronic diseases [2]. Therefore, PA has become a key element of national and international health promotion policies [3–5]. Current recommendations state that adults should avoid inactivity and that substantial health benefits could be obtained from accumulating 150 minutes/week of moderate intensity or 75 min/week of vigorous intensity aerobic activity, in bouts ≥10 min; additional health benefits could be obtained by increasing to 300 minutes/week of moderate intensity or 150 min/week of vigorous intensity aerobic activity. In addition, muscle-strengthening activities of moderate to high intensity should be performed at least 2 days/week.

A significant proportion of the population does not achieve these goals, and both individual and population strategies have been proposed to increase PA [6,7]. An important element of these strategies is the monitoring of their effectiveness by assessing changes in PA practices and behaviors at the individual and population level. Ideally, this assessment should include four dimensions (mode or type of activity, frequency, duration, and intensity) and four domains (occupational, domestic, transportation, and leisure time) [8]. Moreover, sedentary behavior—as differentiated from physical inactivity—has been associated with higher risk of cardiovascular disease [9] and should be assessed as well.

A recently published guide to the assessment of PA states that questionnaires still have a predominant role, but the burden to participants must be low and the evaluation must be completed quickly, inexpensively, and within a single time-point [8]. Several questionnaires are available, but not all of them cover all PA dimensions and domains, as well as sedentary behavior; when all aspects are covered, the time required for administration is burdensome. One of the most widely used instruments is the Minnesota leisure time PA questionnaire (MLTPAQ) [10]; however, one of its main limitations is the high level of burden to the participant and the interviewer. A valid short questionnaire could be very useful both in epidemiological studies and in the clinical setting to characterize physical activity practices and behaviors [8].

The present study aimed to develop and validate a short questionnaire (the REGICOR questionnaire), applicable to clinical settings and epidemiological research and covering all four dimensions (type of activity, frequency, duration, and intensity) and two of the four domains (occupational and leisure time) of PA, as well as sedentary behavior.

## Materials and Methods

### I.-Questionnaire development

#### Design and participants

The long-term REGICOR (Registre Gironi del Cor) project has several different components. This study used data from a population-based cross-sectional study that recruited 6,352 individuals aged 35 to 79 years in Girona (northern Catalonia, Spain) in 2003–2006. The detailed methodology of the study has been described elsewhere [11]. In summary, a random sample of participants from the city of Girona (approximately 70 000 inhabitants) and three surrounding rural towns were invited to participate; the response rate was 73.8%. The study protocol was approved by the Parc Salut MAR (PSMAR) Ethics Committee, was conducted according to the principles expressed in the Declaration of Helsinki and all the participants signed an informed consent.

#### Physical activity questionnaire

The validated Spanish version of the MLTPAQ was administered to all the participants by a trained interviewer [12,13]. Initially, participants were given a list of 67 suggested activities and asked to mark those they had performed during the last year. The interviewer collected information on the number of times each PA was performed and the average time expended each time. Each PA has an intensity code based on the rate of energy expenditure [14]. The questionnaire allows estimation of the total energy expenditure in leisure-time PA, which can also be classified according to intensity (light, moderate, or vigorous).

#### Statistical analysis

A multiple linear regression analysis was performed to identify activities that explained most of the variability of total energy expenditure in leisure time PA. Assumptions on normal distribution of residuals, linear relationship, and homoscedasticity were tested.

#### Questionnaire development

Once the activities that explained most of the population variability of energy expenditure in PA had been identified, an expert committee developed a questionnaire including those activities that would collect information on three of the four key dimensions: type of activity, frequency, duration. The questionnaire was designed to be administered by trained personnel. Definitions of intensity levels were obtained from the most recent (2011) compendium [15]. An algorithm to estimate energy expenditure from light, moderate, and vigorous intensity and total PA was defined and this estimation was considered the main construct of the questionnaire.

Two additional questions designed to capture sedentary behavior asked the number of hours spent watching TV, playing with some type of game console, or playing/working on a computer on a usual day a) during the week and b) on a weekend. These questions were used to estimate the number of weekly hours of sedentary behavior, considered an additional main construct of the questionnaire. Finally, a question related to occupational PA was included, with a choice to be made from 6 categories.

### II.-Questionnaire validation

#### Study design and participants

A study with a cross-sectional component to assess questionnaire validity and a longitudinal component to assess questionnaire reliability and sensitivity to detect changes was designed. A convenience sample of participants recruited in three primary care centers to represent the usual patients of the health care system was stratified by age group (35–54 years and 55–74 years) and sex. The aim was to include 30 individuals in each of the four strata (n = 120). On the selected recruitment days, two patients were randomly selected at each primary care center and were invited to participate. If a selected patient was not willing to participate or met any exclusion criteria, another patient was invited to participate until two patients had been recruited for that day. Participants were excluded if they had been hospitalized within the previous month, had an acute disease, a chronic disease with a life expectancy <1 year, a cognitive or psychiatric disease limiting the administration of questionnaires, or a physical limitation impeding PA.

**Fig 1:**
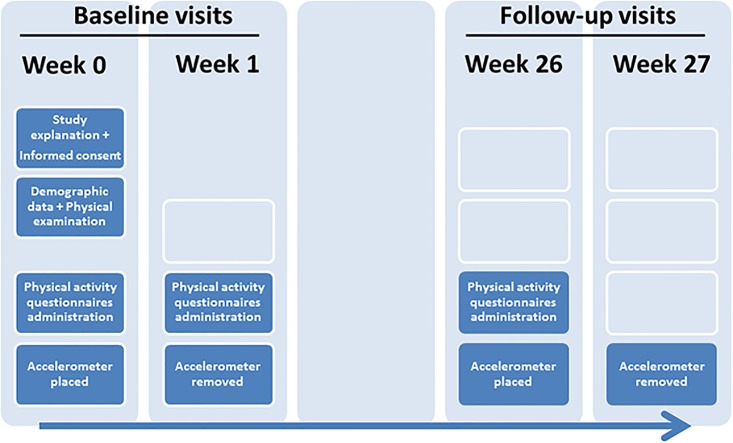
Design of the study: scheduled visits and measurements undertaken at each visit.

The study design is shown in Fig 1. In summary, two initial visits (one week apart) and two follow-up visits (weeks 26 and 27) were defined:

- First visit (Week 0), sociodemographic and clinical variables were collected and an accelerometer was provided, to be worn on the triceps of the left arm for the following week. The Minnesota and REGICOR questionnaires were administered to all participants in random order.
- Second visit (Week 1), the accelerometer was removed and the Minnesota and REGICOR questionnaires were again administered in a random order.
- Third visit (Week 26), the Minnesota and REGICOR questionnaires were administered in a random order. An accelerometer was again provided to be worn on the triceps of the left arm during one week.
- Final visit (Week 27), the accelerometer was removed.

The study protocol was approved by the Parc Salut MAR (PSMAR) Ethics Committee, was conducted according to the principles expressed in the Declaration of Helsinki and all the participants signed an informed consent.

#### Self-reported physical activity

1. The REGICOR questionnaire provides the data needed to estimate the two main constructs: energy expenditure in total PA and in light (< 4 MET), moderate (4–5.5 MET), and vigorous (≥ 6 MET) PA intensity, and the number of weekly hours of sedentary behavior. The questionnaire also provides categorical information about PA at work or in everyday life.
2. The MLTPAQ provides an estimation of the energy expenditure in total PA and in light, moderate and vigorous intensity.

For validation purposes, the energy expenditure related to swimming and biking was excluded, as these activities are not properly registered by the accelerometer we used.

#### Objective physical activity reporting

The SenseWear Pro3 Armband monitor (SWA, Body Media, Pittsburgh, PA, USA), a wireless multisensor activity monitor, integrates motion data from a two-axis accelerometer along with several other physiological sensors (heat flux, skin temperature, and galvanic skin response). The monitor was worn on the left upper arm over the triceps muscle for a minimum of 4 consecutive days and more than 80% of the daytime hours (from 8 am to 10 pm). The maximum time was 7 consecutive days, both daytime and nighttime. Data from the first recording day were not used for the analysis, as it was a partial day and not representative of the participant’s usual PA. The monitor records minute-by-minute and data were processed using the algorithms available in the software (V.7.0.0.2378). This software calculates the number of daily steps, as well as time (hours/day), intensity (MET·min/day), and energy expenditure in total PA (considering activities with an intensity ≥1.4 MET) and categorized by intensity (light, 1.4–3.5 MET; moderate, 3.6–5.9 MET, and vigorous, ≥ 6 MET). This monitor has been shown to provide a valid and objective measurement of energy expenditure in PA in healthy adults [16]. The total number of steps and the number of steps in light, moderate, and vigorous PA performed in bouts exceeding 3 min and 10 min were considered as the main variables of interest for validation purposes.

#### Statistical analysis

Measurements of PA were highly skewed and a significant proportion of participants presented with 0 values; therefore, we transformed the actual values using the inverse hyperbolic sine function to satisfy linearity and normality assumptions assessed by scatter and normal probability plots (QQ plots). This is similar to the log transformation except for an additional capacity to treat the 0 value.

We used the intraclass correlation coefficient to assess the test-retest reliability. To assess the validity of the self-reported PA questionnaire, we calculated the Spearman correlation coefficient between the estimation of the questionnaire and that of the accelerometer. We also used a measurement-error model [17] to calculate the validity coefficient, as recently proposed by Lim et al [18]. To evaluate the sensitivity of the questionnaire to detect changes, we calculated the Spearman correlation coefficient between changes in PA observed with the questionnaire and with the accelerometer between the baseline and the 4-month follow-up visit. All analyses were performed using the R statistical package (version 3.1.0) [19]. A p-value <0.05 was considered statistically significant.

## Results

### A.-Questionnaire development

Six types of PA individually explained more than 5% and globally accounted for 87% of the variability in total PA energy expenditure estimated with the MLTPAQ: walking, brisk walking, gardening, walking trails, climbing stairs, and sport activities (Table 1). Two-part questions (monthly frequency and average daily duration of each activity) were developed to collect information related to the practice of these PA.

**Table 1:** Activities explaining most of the variability of physical activity practice at the population level estimated by the Minnesota Leisure Time Physical Activity Questionnaire.

Physical activity	Change in R^2^	R^2^
**Gardening**	0.26	0.26
**Sport activities**	0.25	0.51
**Climbing stairs**	0.11	0.62
**Walking**	0.11	0.73
**Walking trails**	0.08	0.81
**Brisk walking**	0.06	0.87

To estimate total energy expenditure in leisure time PA, an intensity code was assigned to each type of PA [16]: walking (17270 code: 4 MET), brisk walking (17220 code: 5 MET), gardening (80050 code: 5 MET), walking trails (17080 code: 6 MET), climbing stairs (17130 code: 8 MET), and any sport activity (10 MET) such as swimming, football, gym, etc. An algorithm was defined to estimate energy expenditure in PA: the intensity code was multiplied by the monthly frequency and daily average length of each activity to calculate light, moderate and vigorous intensity and total energy expenditure (S1 File).

We also incorporated two questions about sedentary behavior to elicit information about the number of hours spent watching TV or playing/working on consoles/computers on a typical workday and a typical leisure day. Finally, occupational PA was recorded, based on a single categorical question with six potential activity categories.

The final version of the questionnaire and the algorithm to estimate leisure time energy expenditure in PA is included, with a translation, in the Appendix.

The questionnaire was designed to be administered by trained personnel. They usually needed less than 4 minutes to collect the required information.

### B.-Questionnaire validation

Finally, 114 individuals participated in the validation study (raw data provided in the files of supporting information that accompany the manuscript). The sociodemographic and clinical characteristics of the participants are shown in Table 2.

**Table 2:** Sociodemographic and clinical characteristics of study participants.

		All	Men	Women	35–54 y	55–74 y
		(n = 114)	(n = 51)	(n = 63)	(n = 57)	(n = 57)
**Women, n (%)**		63 (55.3)	---	---	33 (57.9)	30 (52.6)
Age, years*		54.5 (12.1)	54.8 (12.6)	54.2 (11.8)	44.2 (6.2)	64.8 (6.6)
**Education, n (%)**						
	University	34 (29.8)	17 (33.3)	17 (27.0)	21 (36.8)	13 (22.8)
	Secondary	29 (25.4)	11 (21.6)	18 (28.6)	17 (29.8)	12 (21.1)
	Primary	51 (44.7)	23 (45.1)	28 (44.4)	19 (33.3)	32 (56.1)
**BMI, kg/m**^2^*		27.3 (5.0)	28.6 (4.6)	26.2 (5.1)	25.5 (5.1)	29.0 (5.2)
Waist, cm*		95 (14.8)	102 (12.9)	91 (14.6)	90 (13.1)	101 (14.5)
**Smoking, n (%)**						
	Current	36 (21.6)	19 (37.3)	17 (27.0)	23 (40.4)	13 (22.8)
	Former	32 (28.1)	16 (31.4)	16 (25.4)	14 (24.6)	18 (31.6)
	Never	46 (40.4)	16 (31.4)	30 (47.6)	20 (35.1)	26 (45.6)
**Hypertension, n (%)**		40 (35.1)	21 (41.2)	19 (30.2)	7 (12.3)	33 (57.9)
SBP, mmHg*		129 (16.9)	136 (17.2)	124 (14.9)	124 (14.5)	135 (17.4)
DBP, mmHg*		76 (10.4)	78 (9.9)	74 (10.6)	76 (10.3)	77 (10.7)
Heart rate, beats/min*		75 (11.5)	75 (12.9)	75 (10.4)	75 (11.0)	75 (12.2)
**Diabetes, n (%)**		21 (18.4)	15 (29.4)	6 (9.5)	4 (7.0)	17 (29.8)
**Dyslipidemia, n (%)**		41 (36.0)	21 (41.2)	20 (31.7)	16 (28.1)	25 (43.9)

*mean (standard deviation); BMI = Body mass index; SBP = Systolic blood pressure; DBP = Diastolic blood pressure.

Distribution of PA according to the questionnaires and the accelerometer is shown in Table 3.

**Table 3:** Distribution of study participants’ physical activity estimations obtained with the questionnaires and with the accelerometer.

	Percentile 5	Percentile 25	Median	Percentile 75	Percentile 95
**Minnesota Leisure Time Physical Activity Questionnaire (METs·min/week)**					
**Total**	732	1480	2490	4407	6877
**Light intensity**	3	350	691	1490	3989
**Moderate intensity**	0	121	503	1314	3395
**Vigorous intensity**	0	82	504	1270	3670
**REGICOR Questionnaire (METs·min/week)**					
**Total**	662	1369	2664	4166	6811
**Light intensity**	0	336	839	1678	4490
**Moderate intensity**	0	0	332	1262	3528
**Vigorous intensity**	0	23	283	1335	3759
**Accelerometer (steps/day)**					
**Total in 3-min bouts**	2329	5226	7664	10202	14280
**Total in 10-min bouts**	1069	3299	5602	8191	11901
**Light intensity in 3-min bouts**	893	2014	2870	3888	6055
**Light intensity in 10-min bouts**	750	1870	2823	4134	7230
**Moderate intensity in 3-min bouts**	683	2206	4072	6904	9638
**Moderate intensity in 10-min bouts**	3	862	2049	4229	7455
**Vigorous intensity in 3-min bouts**	0	0	0	15	549
**Vigorous intensity in 10-min bouts**	0	0	0	0	149

#### Reliability

The intraclass correlation coefficients (ICCs) in the estimation of energy expenditure in leisure time PA (total, light, moderate, and vigorous intensity) of the two questionnaires (MLTPAQ and REGICOR) administered in a one-week interval are shown in Table 4.

**Table 4:** Reliability of the questionnaires administered in a one-week interval, assessed by the intraclass correlation coefficient.

	MLTPAQ	REGICOR	Sedentary behavior
**Total PA**	0.846 (0.784; 0.891)	0.823 (0.753; 0.875)	0.908 (0.867; 0.937)
**Light intensity PA**	0.861 (0.805; 0.902)	0.809 (0.734; 0.864)	---
**Moderate intensity PA**	0.835 (0.770; 0.883)	0.792 (0.712; 0.852)	---
**High intensity PA**	0.941 (0.915; 0.959)	0.948 (0.925; 0.964)	---

MLTPAQ = Minnesota Leisure Time Physical Activity Questionnaire; PA: Physical Activity.

The reliability was high for both questionnaires, with ICC ranging from 0.792 to 0.948. The estimated number of hours of sedentary behavior also showed a high reliability (ICC = 0.908). These results were similar when the sample was stratified by sex and by age group (S1 Table).

#### Validity

The Spearman correlation coefficients and the validity coefficients observed between the two questionnaires (energy expenditure in PA: total, light, moderate, and vigorous) and the accelerometer data (total number of steps and the number of steps in light, moderate, and vigorous PA performed in bouts exceeding 3 min and 10 min) are shown in Table 5.

**Table 5:** Spearman correlation and validity coefficients between energy expenditure in physical activity estimated with the questionnaires and accelerometer data.

	MLTPAQ	REGICOR
**Spearman correlation coefficient (p-value)**		
**3-min bouts**		
**Total PA**	0.292 (0.002)	0.355 (<0.001)
**Light intensity PA**	0.022 (0.813)	-0.022 (0.817)
**Moderate intensity PA**	0.244 (0.009)	0.404 (<0.001)
**High intensity PA**	0.238 (0.011)	0.259 (<0.001)
**10-min bouts**		
**Total PA**	0.287 (<0.001)	0.385 (<0.001)
**Light intensity PA**	0.102 (0.282)	0.069 (0.464)
**Moderate intensity PA**	0.238 (0.011)	0.381 (<0.001)
**High intensity PA**	0.132 (0.163)	0.080 (0.398)
**Validity coefficient (95% confidence interval)**		
**3-min bouts**		
**Total PA**	0.198 (0.091; 0.305)	0.335 (0.092; 0.578)
**Light intensity PA**	0.045 (-0.222; 0.312)	0.016 (-0.262; 0.294)
**Moderate intensity PA**	0.134 (-0.193; 0.461)	0.506 (0.256; 0.756)
**High intensity PA**	0.486 (0.173; 0.799)	0.545 (0.221; 0.869)
**10-min bouts**		
**Total PA**	0.280 (0.179; 0.381)	0.501 (0.279; 0.723)
**Light intensity PA**	0.015 (-0.174; 0.204)	0.005 (-0.203; 0.213)
**Moderate intensity PA**	0.281 (-0.094; 0.656)	0.542 (0.205; 0.879)
**High intensity PA**	0.229 (-0.045; 0.503)	0.118 (-0.274; 0.510)

MLTPAQ = Minnesota Leisure Time Physical Activity Questionnaire; PA: Physical Activity.

In general, the validity of REGICOR questionnaire results was slightly better, although both questionnaires were correlated with total and moderate PA estimated with the accelerometer and neither was correlated with the accelerometer's estimation of energy expenditure in light intensity PA. Only the REGICOR questionnaire results correlated with vigorous intensity PA estimated with the accelerometer, and only when bouts longer than 3 min were considered. The Spearman correlation coefficient between weekly hours of sedentary behaviors estimated with the REGICOR questionnaire and those estimated with the accelerometer was 0.244 (p-value = 0.020).

The validity coefficient showed better results than those obtained with the Spearman correlation for total, moderate, and vigorous physical activity. The results of the REGICOR questionnaire were again better than the MLTPAQ, but the pattern was similar to those observed with the Spearman correlation. The MLTPAQ’s estimation of vigorous intensity PA showed a significant validity coefficient with the accelerometer, considering bouts longer than 3 min. Results were similar when the sample was stratified by sex, and slightly better validity was observed in the group aged 55–74 years, compared to the younger group (S2 Table).

#### Sensitivity to detect changes

Changes in PA between the baseline and the final visit as estimated with the questionnaires and observed with the accelerometer are shown in S3 Table. The REGICOR questionnaire was able to detect changes in moderate and vigorous intensity PA (Table 6). The MLTPAQ did not detect these changes and showed an inverse correlation for changes in vigorous intensity PA (Table 6).

**Table 6:** Sensitivity to detect changes in physical activity of the Minnesota Leisure Time Physical Activity Questionnaire and the REGICOR questionnaire, assessed by the Spearman correlation coefficient between changes estimated by the questionnaires and changes detected by the accelerometer.

	Changes estimated with the MLTPAQ		Changes estimated with the REGICOR	
Changes detected with the accelerometer	Spearman correlation coefficient	p-value	Spearman correlation coefficient	p-value
**3-min bouts**				
**Total PA**	0.198	0.079	0.091	0.388
**Light intensity PA**	0.071	0.532	0.030	0.777
**Moderate intensity PA**	0.158	0.163	0.344	0.001
**Vigorous intensity PA**	-0.063	0.576	0.275	0.008
**10-min bouts**				
**Total PA**	0.157	0.164	0.088	0.404
**Light intensity PA**	0.093	0.412	0.050	0.634
**Moderate intensity PA**	0.098	0.388	0.264	0.011
**Vigorous intensity PA**	-0.170	0.132	-0.015	0.887

MLTPAQ = Minnesota Leisure Time Physical Activity Questionnaire; PA: Physical Activity.

## Discussion

The present study developed a short questionnaire that accounts for most of the variability in leisure time PA at population level (healthy adults, Girona provice, northern Catalonia), as estimated by the much more extensive and labor-intensive MLTPAQ. The short REGICOR questionnaire also records information about sedentary behavior and occupational PA. The questionnaire is a reliable and valid method to estimate moderate and vigorous intensity leisure time PA and sedentary behavior, and sensitive to detect changes in moderate and vigorous intensity leisure time PA.

The REGICOR questionnaire includes six two-part questions that collect information on the four dimensions of PA (type of activity, frequency, duration, and intensity). Activities were clustered into six groups: walking, brisk walking, hiking, climbing stairs, gardening, and indoor or outdoor exercise or sports. The REGICOR questionnaire provides an estimation of energy expenditure in leisure time PA, and also classifies this expenditure according to light, moderate and vigorous PA. Similar to the classic MLTPAQ, the short questionnaire focuses mainly on one PA domain: leisure time. However, it collects information on walking, the main mode of physically active transportation, and includes a categorical classification of occupational PA.

The REGICOR questionnaire has a very high reliability, ranging from 0.79 for moderate intensity PA to 0.95 for vigorous intensity, similar to that observed for the MLTPAQ in this and in previous studies [20] and reported for other questionnaires [21,22]. The reliability for sedentary behavior is also high.

To assess the validity and sensitivity of the questionnaire to detect changes, we chose the accelerometer as an objective measurement of PA. There is an open debate about the direct comparison between the accelerometers and self-reported data [23,24], as these assessment methods are not equivalent. Accelerometer-based monitors quantify acceleration from body motion at a fixed point of the body (in our case, the upper arm) over short time periods. On the other hand, questionnaires register self-reported PA and time reportedly spent on concrete behaviors is used to quantify it. This self-report behavior is a proxy of body motion but also incorporates psychosocial and environmental elements, activity purpose, perceived time and intensity of the effort [24]–and usually overestimates PA [25]. Nonetheless, the comparison between these two methods is frequently used to assess the validity of new questionnaires or of classic questionnaires in specific populations [22] as accelerometer-based measurements are objective and independent of bias associated with self-reporting. To compare these two distinct approaches to PA measurement, we used the direct estimation of the amount of PA provided by the accelerometer-based monitor and the questionnaire: daily step counts and energy expenditure in MET·min/day, respectively. To avoid introducing new assumptions into the estimations of PA, we did not incorporate other energy expenditure measurements such as calories/day [26]. Moreover, we used step counts in bouts longer than 3 min and 10 min to exclude brief, episodic body motions registered by the accelerometer.

The correlation between the two PA measurements was low to moderate but concordant to that reported in other studies [22]. In this study, we also used the validity coefficient proposed by Lim et al [19]; these coefficients are slightly higher but concordant with the Spearman coefficients. This low-to-moderate correlation could be related to the recall bias associated with self-reporting, to accelerometer measurement errors [27], and to the fact that the two instruments are not measuring exactly the same construct. These results point out some of the limitations of the questionnaires when compared to objective measures of PA but also the need to develop better accelerometer algorithms to improve the differentiation between modes and intensities of PA. The main implications in epidemiological research include the validity of PA assessment when using questionnaires, the direction of the bias in self-reported PA and the effects of this measurement error on the association under study. Some authors have suggested using accelerometer-based PA measurements in a subsample of participants to quantify and determine the direction of the bias in self-reported PA allowing for correction of the bias [19] using statistical techniques such as regression calibration methods [28].

Overall, the correlation coefficients were slightly better for the short REGICOR questionnaire than for the classic MLTPAQ. Globally, the analysis indicates that the validity of REGICOR questionnaire results, obtained at substantially lower burden for the participant and the interviewer, is similar to that of MLTPAQ results. Moreover, we observed that the short questionnaire had an acceptable correlation with the accelerometer for moderate and vigorous intensity but not for light intensity PA. The correlation for vigorous intensity was lower than for moderate PA, and was only observed when bouts longer than 3 min were considered (i.e., not for bouts longer than 10 min). Three possible explanations should be considered: i) in the shorter questionnaire, a fixed energy expenditure was assigned to all exercise and sports-related activities (10 METs), which could introduce some error by not considering variability in the intensity of vigorous exercise; ii) several studies have shown that accelerometers are not as valid in registering very vigorous PA [24]; and iii) the practice of vigorous PA in bouts longer than 10 min was very infrequent in the population included in this validation study. The lack of correlation between self-reported and objectively registered light intensity PA could be related to the lack of attention to household activities in the shorter questionnaire or to the possibility that some individuals consider this type of PA unimportant and underreport it. This limitation could be considered irrelevant, as current recommendations focus on moderate to vigorous PA [3–5]; however, some studies have shown an association between light intensity PA, such as walking, and mortality or coronary heart disease risk, especially in older individuals [28–31]. Therefore, and although these studies used questionnaires to assess light intensity PA, the association between the questionnaire light intensity PA estimation and health outcomes should be evaluated with caution.

Finally, we observed that the REGICOR questionnaire is sensitive to detect changes in moderate and vigorous PA. Few questionnaires have demonstrated good sensitivity to detect changes [32–34]. The MLTPAQ was not able to detect such changes in our study, although we must take into consideration that this questionnaire was designed to assess PA performed in the previous year. Changes during a shorter period, such as in our study (4 months), could be diluted and underestimated.

Among the strengths of the study, we would note that the short questionnaire developed by the REGICOR team covers all the domains of physical activity. Although it is mainly focused on the leisure time dimension, it includes walking as both a mode of transportation and as a leisure activity. The development of the questionnaire was based on data provided by more than 6,000 individuals representative of the general population. The questionnaire has a low cost burden for both participants/patients and researchers/medical personnel, but provides a valid estimation of moderate and vigorous energy expenditure in PA. The convenience sampling and the results of the stratified analyses indicate the validity of the questionnaire in a 40-year age range and in both men and women.

The study limitations include the development of the questionnaire based on the selection of the activities that explained most of the variance of total physical activity and on expert criteria based on PA dimensions and domains; the patient perspective was not considered [35]. In addition, the questionnaire was developed in a Southern European Mediterranean population based on PA that explains most of the variability observed at the population level. Other PA not included in this questionnaire could be relevant in other populations, such as bicycling to work or for leisure. However, bicycling to work or for leisure has the same intensity code as walking and could be included in this category in populations in which this PA is common. Finally, the validity was tested only in the population aged 35 to 74 years, and the questionnaire asks about PA in the previous month, which might not be representative of regular practices and behaviors.

In conclusion, we developed a short PA questionnaire, mainly but not solely focused on one domain (leisure time), that collects information on the four dimensions of PA and is reliable, valid, and sensitive to detect changes in moderate and vigorous intensity. This questionnaire has a low burden of time and effort and could be used in daily clinical practice and epidemiological studies.

## Supporting Information

S1 Table Reliability of the questionnaires administered in a one-week interval assessed by the intraclass correlation coefficient across sex and age-groups. (DOCX)

S2 Table Spearman correlation coefficients between questionnaire-based estimates of energy expenditure in physical activity and accelerometer data, overall by time segment and dichotomized by sex and age group. (DOCX)

S3 Table Changes in physical activity practices and behaviors between baseline and follow-up visits estimated with the questionnaires and detected by the accelerometer. (DOCX)

S1 File I.-The validated Spanish language version of the questionnaire is provided. II.-The English language version of the questionnaire is provided. III.-The algorithms to estimate leisure time energy expenditure in physical activity are provided.
(DOCX)
