# Supplementary material for: Validation of the Regicor Short Physical Activity Questionnaire for the Adult Population
Source: PLoS One. 2017 Jan 13;12(1):e0168148. doi: 10.1371/journal.pone.0168148 (PMC5234797; doi:10.1371/journal.pone.0168148)
Supplement: S1 Table — (DOCX) [file pone.0168148.s001.docx]

**S1 Table**. Reliability of the questionnaires administered in a one-week interval assessed by the intraclass correlation coefficient across sex and age groups.

|  | **MLTPAQ** | **REGICOR** | **Sedentary behavior** |
| --- | --- | --- | --- |
| **Men (n=51)** | | | |
| Total PA | 0.800 (0.674; 0.881) | 0.770 (0.631; 0.862) | 0.918 (0.856; 0.954) |
| Light intensity PA | 0.859 (0.767; 0.917) | 0.780 (0.645; 0.868) | --- |
| Moderate intensity PA | 0.858 (0.764; 0.916) | 0.812 (0.693; 0.888) | --- |
| Vigorous intensity PA | 0.907 (0.843; 0.946) | 0.933 (0.886; 0.961) | --- |
| **Women (n=63)** | | | |
| Total PA | 0.880 (0.810; 0.926) | 0.868 (0.790; 0.919) | 0.900 (0.838; 0.939) |
| Light intensity PA | 0.847 (0.760; 0.905) | 0.843 (0.752; 0.903) | --- |
| Moderate intensity PA | 0.806 (0.700; 0.878) | 0.779 (0.657; 0.861) | --- |
| Vigorous intensity PA | 0.954 (0.925; 0.972) | 0.951 (0.920; 0.970) | --- |
| **35-54 years (n=57)** | | | |
| Total PA | 0.781 (0.653; 0.865) | 0.885 (0.813; 0.930) | 0.928 (0.880; 0.958) |
| Light intensity PA | 0.668 (0.496; 0.790) | 0.581 (0.381; 0.730) | --- |
| Moderate intensity PA | 0.813 (0.703; 0.885) | 0.822 (0.716; 0.891) | --- |
| Vigorous intensity PA | 0.922 (0.871; 0.953) | 0.983 (0.972; 0.990) | --- |
| **55-74 years (n=57)** | | | |
| Total PA | 0.890 (0.821; 0.934) | 0.758 (0.620; 0.851) | 0.882 (0.802; 0.931) |
| Light intensity PA | 0.896 (0.829; 0.937) | 0.842 (0.744; 0.904) | --- |
| Moderate intensity PA | 0.853 (0.764; 0.911) | 0.766 (0.631; 0.856) | --- |
| Vigorous intensity PA | 0.961 (0.936; 0.977) | 0.905 (0.843; 0.943) | --- |

MLTPAQ=Minnesota Leisure Time Physical Activity Questionnaire; PA: Physical Activity.
