# Supplementary material for: Validation of the Regicor Short Physical Activity Questionnaire for the Adult Population
Source: PLoS One. 2017 Jan 13;12(1):e0168148. doi: 10.1371/journal.pone.0168148 (PMC5234797; doi:10.1371/journal.pone.0168148)
Supplement: S2 Table — (DOCX) [file pone.0168148.s002.docx]

**S2 Table.** Spearman correlation coefficients between questionnaire-based estimates of energy expenditure in physical activity and accelerometer data, across sex and age groups.

|  | **MLTPAQ** | **REGICOR** |
| --- | --- | --- |
| **Men (n=51)** | | |
| **3-min bouts** | | |
| Total PA | 0.303 (0.031) | 0.342 (0.014) |
| Light intensity PA | 0.112 (0.433) | -0.043 (0.765) |
| Moderate intensity PA | 0.047 (0.742) | 0.283 (0.044) |
| Vigorous intensity PA | 0.233 (0.101) | 0.305 (0.030) |
| **10-min bouts** | | |
| Total PA | 0.339 (0.015) | 0.409 (<0.001) |
| Light intensity PA | 0.219 (0.122) | 0.139 (0.329) |
| Moderate intensity PA | 0.090 (0.530) | 0.322 (0.021) |
| Vigorous intensity PA | 0.166 (0.244) | 0.289 (0.040) |
| **Women (n=63)** | | |
| **3-min bouts** | | |
| Total PA | 0.305 (0.015) | 0.358 (<0.001) |
| Light intensity PA | 0.059 (0.647) | 0.080 (0.535) |
| Moderate intensity PA | 0.401 (<0.001) | 0.568 (<0.001) |
| Vigorous intensity PA | 0.257 (0.042) | 0.256 (0.043) |
| **10-min bouts** | | |
| Total PA | 0.288 (0.023) | 0.362 (<0.001) |
| Light intensity PA | 0.092 (0.472) | 0.079 (0.541) |
| Moderate intensity PA | 0.370 (<0.001) | 0.488 (<0.001) |
| Vigorous intensity PA | 0.152 (0.235) | -0.191 (0.134) |
| **35-54 years (n=57)** | | |
| **3-min bouts** | | |
| Total PA | 0.188 (0.162) | 0.378 (<0.001) |
| Light intensity PA | 0.195 (0.147) | 0.140 (0.299) |
| Moderate intensity PA | 0.173 (0.199) | 0.440 (<0.001) |
| Vigorous intensity PA | 0.301 (0.023) | 0.163 (0.225) |
| **10-min bouts** | | |
| Total PA | 0.155 (0.250) | 0.380 (<0.001) |
| Light intensity PA | 0.239 (0.073) | 0.143 (0.287) |
| Moderate intensity PA | 0.166 (0.216) | 0.384 (<0.001) |
| Vigorous intensity PA | 0.109 (0.419) | -0.008 (0.952) |
| **55-74 years (n=57)** | | |
| **3-min bouts** | | |
| Total PA | 0.430 (<0.001) | 0.430 (<0.001) |
| Light intensity PA | -0.104 (0.439) | -0.104 (0.443) |
| Moderate intensity PA | 0.297 (0.025) | 0.409 (<0.001) |
| Vigorous intensity PA | 0.002 (0.987) | 0.335 (0.011) |
| **10-min bouts** | | |
| Total PA | 0.406 (<0.001) | 0.440 (<0.001) |
| Light intensity PA | -0.016 (0.908) | 0.008 (0.955) |
| Moderate intensity PA | 0.313 (0.018) | 0.410 (<0.001) |
| Vigorous intensity PA | 0.042 (0.756) | 0.181 (0.177) |

MLTPAQ=Minnesota Leisure Time Physical Activity Questionnaire; PA: Physical Activity.
