# Supplementary material for: Validation of the Regicor Short Physical Activity Questionnaire for the Adult Population
Source: PLoS One. 2017 Jan 13;12(1):e0168148. doi: 10.1371/journal.pone.0168148 (PMC5234797; doi:10.1371/journal.pone.0168148)
Supplement: S3 Table — (DOCX) [file pone.0168148.s003.docx]

**S3 Table.** Changes in physical activity practice between baseline and follow-up visits detected by the accelerometer and estimated with the questionnaires. Values are transformed using the inverse hyperbolic sine function and shown as median (quartile 1; quartile 3).

|  | **Total PA** | **Light PA** | **Moderate PA** | **Vigorous PA** |
| --- | --- | --- | --- | --- |
| **Accelerometer 3-min bouts** | 0.032 (-0.246; 0.243) | -0.036 (-0.261; 0.227) | 0.040 (-0.252; 0.343) | 0 (0; 0.895) |
| **Accelerometer 10-min bouts** | 0.081 (-0.314; 0.367) | 0.066 (-0.375; 0.269) | 0.015 (-0.367; 0.636) | 0 (0; 0) |
| **MLTPAQ** | 0.021 (-0.333; 0.395) | -0.069 (-0.861; 0.242) | -0.102 (-0.981; 0.751) | 0.328 (-0.568; 1.180) |
| **Regicor questionnaire** | -0.027 (-0.294; 0.238) | -0.032 (-0.946; 0.599) | 0 (-0.067; 0.323) | 0 (-1.970; 1.540) |
