# Supplementary material for: Validation of the Regicor Short Physical Activity Questionnaire for the Adult Population
Source: PLoS One. 2017 Jan 13;12(1):e0168148. doi: 10.1371/journal.pone.0168148 (PMC5234797; doi:10.1371/journal.pone.0168148)
Supplement: S1 File — I.-The validated Spanish language version of the questionnaire is provided. II.-The English language version of the questionnaire is provided. III.-The algorithms to estimate leisure time energy expenditure in physical activity are provided. (DOCX) [file pone.0168148.s004.docx]

**Supplementary Material**

**I.-The validated Spanish-language version of the questionnaire is provided below.**

Actividad física en el tiempo libre

A continuación le preguntaremos sobre actividades físicas realizadas en un mes convencional.

En un mes normal:

1.a. ¿Cuántos días pasea o camina tranquilamente?† |__|__| días

1.b. De promedio, ¿cuántos minutos al día? |__|__|__| minutos/día

2.a. ¿Cuántos días camina deprisa? |__|__| días

2.b. De promedio, ¿cuántos minutos al día? |__|__|__| minutos/día

3.a. ¿Cuántos días camina campo a través, va de excursión? |__|__| días

3.b. De promedio, ¿cuántos minutos al día? |__|__|__| minutos/día

4.a. ¿Cuántos días sube escaleras? |__|__| días

4.b. De promedio, ¿cuántos pisos al día? |__|__|__| pisos/día

5.a. ¿Cuántos días trabaja en el huerto o en el jardín? |__|__| días

5.b. De promedio, ¿cuántos minutos al día? |__|__|__| minutos/día

6.a. ¿Cuántos días hace ejercicios, deportes al aire libre o en casa o en el gimnasio? |__|__| días

6.b. De promedio, ¿cuántos minutos al día? |__|__|__| minutos/día

*† NOTA: Incluye caminar al trabajo. También se puede incluir ir en bicicleta al trabajo o pasear en bicicleta.*

Comportamiento sedentario

De promedio, durante un día laborable y fuera del trabajo ¿cuántas horas al día está viendo la televisión/ordenador/videojuegos? |__|__| horas/día

De promedio, durante un día no laborable ¿cuántas horas al día está viendo la televisión/ordenador/videojuegos? |__|__| horas/día

¿Cuántos días trabaja a la semana? |__| días

Actividad física laboral

¿Qué tipo de actividad física realiza en su lugar de trabajo (o en su vida cotidiana) ?

a.-básicamente estoy sentado/a y camino poco (funcionario, administrativo...)

b.-estoy sentado/a pero hago esfuerzos moderados continuos (cajero...)

c.-básicamente estoy de pie sin moverme

d.-camino bastante pero no hago ningún esfuerzo vigoroso (vendedor, comercial...)

e.-camino bastante y hago esfuerzos vigorosos (cartero, transportista...)

f.-básicamente hago esfuerzos vigorosos y de mucha actividad (construcción, cargadores...)

**II.-An English version of the developed and validated short questionnaire is provided below.**

Leisure time physical activity

I am going to ask you some questions about your physical activity during a typical month.

In a typical month:

1.a. How many days do you go for a walk at a slow or normal pace? † |__|__| days

1.b. On average, how many minutes each day? |__|__|__| min/day

2.a. How many days do you walk fast (brisk walking)? |__|__| days

2.b. On average, how many minutes each day? |__|__|__| min/day

3.a. How many days do you walk in the countryside or in the mountains? |__|__| days

3.b. On average, how many minutes each day? |__|__|__| min/day

4.a. How many days do you climb stairs? |__|__| days

4.b. On average, how many floors each day? |__|__|__| floors/day

5.a. How many days do you work in the yard or garden? |__|__| days

5.b. On average, how many minutes each day? |__|__|__| min/day

6.a. How many days do you exercise or play sports at home, outdoors,

or in a gym? |__|__| days

6.b. On average, how many minutes each day? |__|__|__| min/day

*† NOTE: Walking for commuting to work is included. Bicycling for commuting to work or for pleasure could also be included in this item.*

Sedentary behavior

On average during a typical work-day and excluding work time, how many hours do you watch TV or sit at a computer or play video games? |__|__| hours/day

On average during a non-working day, how many hours do you watch TV or sit at a computer or play video games? |__|__| hours/day

How many days per week do you work? |__| days

Occupational physical activity

What type of physical activity do you perform in your occupation (or in your daily life)?

a.- Basically, I’m seated and I walk very little (administrative, ...)

b.- I am seated but I very often perform moderate intensity efforts (cashier...)

c.- Basically, I am standing and I walk very little

d.- I walk a lot but I do not perform vigorous effort (salesperson, shopkeeper...)

e.- I walk a lot and I perform vigorous effort (mail carrier, delivery person...)

f.- Basically, I perform vigorous effort (construction worker...)

**III.-Algorithms to estimate leisure time energy expenditure in physical activity.**

With the REGICOR physical activity questionnaire we can estimate total energy expenditure in leisure time physical activity. Moreover, we can estimate energy expenditure in physical activity based on its intensity: light intensity physical activity (<4 MET), moderate intensity (4 a 5.5 MET) and vigorous intensity (≥6 MET). All estimations are expressed as MET·min/week.

Estimation of energy expenditure in light intensity physical activity (EEPA_light_).

EEPA_light_ = [(Number of days of walking (1.a) x Minutes of walking per day (1.b) x 4 MET) / 4.29]

Estimation of energy expenditure in moderate intensity physical activity (EEPA_mod_).

EEPA_mod_ = [(Number of days of brisk walking (2.a) x Minutes of brisk walking per day (2.b) x 5 MET) / 4.29] + [(Number of days of gardening (5.a) x Minutes of gardening per day (5.b) x 5 MET) / 4.29]

Estimation of energy expenditure in vigorous intensity physical activity (EEPA_vig_).

EEPA_vig_ = [(Number of days of walking in the countryside (3.a) x Minutes of walking in the countryside per day (3.b) x 6 MET) / 4.29] + [(Number of days of climbing stairs (4.a) x Number of floors climbed per day (4.b) x 3.5 MET) / 4.29] + [(Number of days of exercising/sport practice (6.a) x Minutes of exercise per day (6.b) x 11) / 4.29]

Estimation of total energy expenditure in physical activity (EEPA_Total_).

EEPA_Total_ = EEPA_ligth_ + EEPA_mod_ + EEPA_vig_
